# Supplementary material for: Associations of Cooking Skill with Social Relationships and Social Capital among Older Men and Women in Japan: Results from the JAGES
Source: Int J Environ Res Public Health. 2023 Mar 6;20(5):4633. doi: 10.3390/ijerph20054633 (PMC10002414; doi:10.3390/ijerph20054633)
Supplement: Supplementary file 1 [file ijerph-20-04633-s001.zip › ijerph-2240211-supplementary.pdf]

Table S1. Characteristics of older men and women in Japan by level of cooking skill

	Men (n = 9,551)						Women (n = 11,510)					p-value
	Total		Cooking skill				Total		Cooking skill			
	n	%	High %	Middle %	Low %	p-value	n	%	High %	Middle/Low %	p-value	
Cooking skill												
High	4,952	51.8	100.0	0.0	0.0		10,806	93.9	100.0	0.0		<.001
Middle	3,420	35.8	0.0	100.0	0.0		563	4.9	0.0	80.0		
Low	1,179	12.3	0.0	0.0	100.0		141	1.2	0.0	20.0		
Age (years)												
65–69	2,926	30.6	32.2	29.2	28.5	< .001	3,422	29.7	30.6	17.0	< .001	.19
70–74	2,593	27.1	26.8	28.7	24.4		3,077	26.7	27.2	20.0		
75–79	2,151	22.5	22.9	22.3	21.6		2,623	22.8	23.0	19.9		
≥ 80	1,881	19.7	18.2	19.9	25.4		2,388	20.7	19.3	43.0		
Socio-demographic characteristics												
Education (years)												
Low (≤ 9)	2,813	29.5	29.1	28.5	33.4	.001	4,079	35.4	34.6	47.6	< .001	< .001
Middle (10–12)	3,588	37.6	36.4	39.4	37.3		4,929	42.8	43.3	36.1		
High (≥ 13)	3,063	32.1	33.6	31.2	28.2		2,317	20.1	20.5	13.8		
Other/Missing	87	0.9	0.9	0.9	1.0		185	1.6	1.5	2.6		
Annual income (million yen)												
Low (< 2.00)	3,648	38.2	37.6	38.9	38.7	.12	4,411	38.3	38.1	42.0	< .001	< .001
Middle (2.00–3.99)	3,367	35.3	35.5	35.9	32.6		3,175	27.6	28.1	19.7		
High (≥ 4.00)	947	9.9	10.3	9.2	10.3		916	8.0	8.1	5.4		
Missing	1,589	16.6	16.6	16.0	18.5		3,008	26.1	25.7	32.8		

Marital status												
Married	8,104	84.8	81.3	87.7	91.5	< .001	6,943	60.3	61.4	43.6	< .001	< .001
Widowed	681	7.1	8.7	5.8	4.4		3,448	30.0	29.0	43.9		
Divorced	331	3.5	4.7	2.5	1.2		542	4.7	4.7	5.0		
Never married	267	2.8	3.4	2.5	1.1		332	2.9	2.8	3.7		
Other/Missing	168	1.8	2.0	1.4	1.8		245	2.1	2.0	3.8		
Health status												
Under medical treatment												
Cancer (Yes)	463	4.8	4.9	4.5	5.5	.67	378	3.3	3.2	4.4	.23	< .001
Heart disease (Yes)	1,266	13.3	13.0	13.4	14.0	.90	786	6.8	6.6	9.9	.003	< .001
Stroke (Yes)	404	4.2	3.5	4.5	6.7	< .001	198	1.7	1.6	4.1	< .001	< .001
Diabetes mellitus (Yes)	1,540	16.1	16.6	15.2	17.0	.44	1,097	9.5	9.5	10.4	.74	< .001
Hypertension (Yes)	4,096	42.9	42.1	43.5	44.1	.61	4,790	41.6	41.2	47.3	.006	< .001
Hyperlipidemia (Yes)	965	10.1	10.1	10.4	9.5	.94	1,754	15.2	15.4	12.6	.14	< .001
Depressive symptoms												
No depression (GDS < 5)	6,446	67.5	70.0	65.9	61.6	< .001	7,237	62.9	64.0	45.5	< .001	< .001
Depressive symptoms (GDS ≥ 5)	1,950	20.4	18.4	21.7	25.4		1,943	16.9	16.1	28.6		
Missing	1,155	12.1	11.6	12.5	13.0		2,330	20.2	19.9	26.0		
Prosocial behavior-related personality												
Conversation with young people												
No	2,411	25.2	21.4	27.7	33.9	< .001	1,839	16.0	15.0	31.0	< .001	< .001
Yes	6,979	73.1	76.7	70.9	64.3		9,411	81.8	82.8	65.8		
Missing	161	1.7	1.9	1.3	1.8		260	2.3	2.2	3.3		
Willingness to play a leadership role in a community activity												
Do not want to do this	5,078	53.2	50.0	54.8	61.8	< .001	6,481	56.3	55.8	64.3	< .001	< .001

May do this	3,587	37.6	40.3	36.5	29.1		3,413	29.7	30.4	17.6		
Want to do this	234	2.5	3.0	2.0	1.4		286	2.5	2.6	1.4		
Missing	652	6.8	6.6	6.8	7.6		1,330	11.6	11.2	16.6		
Social relationships												
Neighborhood ties												
High	1,129	11.9	13.2	11.1	9.2	< .001	2,267	20.0	20.3	15.3	< .001	< .001
Middle	4,826	51.1	51.3	51.5	48.8		6,754	59.5	59.9	52.7		
Low	3,494	37.0	35.5	37.4	42.0		2,329	20.5	19.8	32.0		
Eating with friends												
No	9,143	95.7	95.2	96.1	96.8	.03	10,367	90.1	89.8	93.9	< .001	< .001
Yes	408	4.3	4.8	3.9	3.2		1,143	9.9	10.2	6.1		
	mean	SD	mean	mean	mean		mean	SD	mean	mean		
Frequency of meetings with friends (*n*/week)	1.25	1.42	1.33	1.21	1.06	< .001	1.59	1.43	1.62	1.18	< .001	< .001
Number of meetings with friends (*n*/month)	5.37	3.80	5.53	5.34	4.77	< .001	6.04	3.52	6.15	4.40	< .001	< .001
Social capital	*n*	%	%	%	%		*n*	%	%	%		
Civic participation												
No groups	4,530	59.4	57.6	59.6	66.9	< .001	4,078	50.9	49.8	68.5	< .001	< .001
One group	1,282	16.8	17.5	16.9	13.8		1,356	16.9	17.2	13.3		
Two groups	1,071	14.1	14.2	14.3	12.7		1,469	18.3	18.8	11.6		
Three groups	499	6.5	7.0	6.4	5.1		720	9.0	9.3	4.5		
Four groups	174	2.3	2.7	2.0	1.2		299	3.7	3.9	1.7		
Five groups	65	0.9	1.1	0.7	0.3		88	1.1	1.1	0.4		
Social cohesion												

No items	1,283	13.7	14.2	13.0	13.6	.19	1,625	14.7	14.3	21.0	< .001	< .001
One item	1,586	16.9	16.3	17.4	18.3		2,055	18.6	18.5	19.3		
Two items	2,091	22.3	21.7	23.4	21.8		2,179	19.7	19.9	16.5		
Three items	4,403	47.0	47.7	46.2	46.3		5,202	47.0	47.3	43.2		
Reciprocity												
No items	4,945	53.7	49.9	56.0	62.6	< .001	3,058	27.8	26.8	43.4	< .001	< .001
One item	1,179	12.8	13.4	12.8	10.2		1,413	12.8	12.9	12.4		
Two items	2,827	30.7	32.8	29.5	25.3		5,377	48.8	49.6	37.2		
Three items	265	2.9	4.0	1.6	1.9		1,160	10.5	10.8	7.0		

GDS = Geriatric Depression Scale; SD = standard deviation

Table S2. Associations of cooking skill with neighborhood ties, frequent meals with friends, and frequency and number of meetings with friends among older adults in Japan

		Neighborhood ties (ref = low-level ties)		Frequent meals with friends	Frequency of meetings with friends (*n*/week)	Number of meetings with friends (*n*/week)
		Middle-level tie	High-level tie			
		RRR (95% CI)	RRR (95% CI)	OR (95% CI)	coefficient (95% CI)	coefficient (95% CI)
Women						
Cooking skill	Middle/Low	ref	ref	ref	ref	ref
	High	**1.61 (1.34 to 1.94)**	**1.86 (1.44 to 2.41)**	**1.53 (1.11 to 2.11)**	**0.27 (0.16 to 0.38)**	**1.03 (0.76 to 1.30)**
Men						
Cooking skill	Low	ref	ref	ref	ref	ref
	Middle	1.14 (0.98 to 1.32)	1.24 (0.96 to 1.59)	1.03 (0.71 to 1.49)	0.07 (-0.03 to 0.16)	**0.27 (0.03 to 0.51)**
	High	**1.16 (1.01 to 1.35)**	**1.44 (1.13 to 1.83)**	1.06 (0.74 to 1.51)	**0.10 (0.02 to 0.19)**	**0.26 (0.03 to 0.49)**

CI = confidence interval; OR = odds ratio; ref = reference group; RRR = relative risk ratio; SD = standard deviation

Boldface indicates statistical significance ($p < 0.05$).

These models adjusted for age, education, annual normalized household income, marital status, health status (cancer, heart disease, stroke, diabetes, hypertension, hyperlipidemia, and depressive symptoms), and prosocial behavior-related personality.

Table S3. Results of regression analyses of social capital according to the cooking skill among older adults in Japan

		Civic participation	Social cohesion	Reciprocity
		0–5	0–3	0–3
		coefficient (95% CI)	coefficient (95% CI)	coefficient (95% CI)
Women				
Cooking skill	Middle/Low	ref	ref	ref
	High	**0.14 (0.03 to 0.25)**	0.05 (-0.03 to 0.14)	**0.20 (0.13 to 0.28)**
Men				
Cooking skill	Low	ref	ref	ref
	Middle	0.07 (-0.01 to 0.15)	-0.02 (-0.09 to 0.04)	0.03 (-0.03 to 0.09)
	High	**0.08 (0.01 to 0.16)**	-0.05 (-0.12 to 0.01)	**0.10 (0.04 to 0.16)**

CI = confidence interval; ref = reference group

Boldface indicates statistical significance ($p < 0.05$).

These models adjusted for age, education, annual normalized household income, marital status, health status (cancer, heart disease, stroke, diabetes, hypertension, hyperlipidemia, and depressive symptoms), and prosocial behavior-related personality.

Table S4. Associations of gender with neighborhood ties, frequent meals with friends, and frequency and number of meetings with friends among older adults in Japan

	Neighborhood ties (ref = low-level ties)		Eating with friends	Frequency of meetings with friends (*n*/week)	Number of meetings with friends (*n*/week)
	Middle-level ties	High-level ties			
	RRR (95% CI)	RRR (95% CI)	OR (95% CI)	coefficient (95% CI)	coefficient (95% CI)
Gender					
Men	ref	ref	ref	ref	ref
Women	**2.10 (1.97 to 2.24)**	**3.01 (2.76 to 3.29)**	**2.47 (2.20 to 2.78)**	**0.34 (0.30 to 0.38)**	**0.67 (0.57 to 0.78)**

CI = confidence interval; OR = odds ratio; ref = reference group; RRR = relative risk ratio; SD = standard deviation

Boldface indicates statistical significance ($p < 0.05$).

Table S5. Results of regression analyses of social capital according to gender among older adults in Japan

	Civic participation	Social cohesion	Reciprocity
	0–5	0–3	0–3
	coefficient (95% CI)	coefficient (95% CI)	coefficient (95% CI)
Gender			
Men	ref	ref	ref
Women	**0.23 (0.19 to 0.27)**	**-0.04 (-0.07 to -0.01)**	**0.11 (0.10 to 0.13)**

CI = confidence interval; ref = reference group

Boldface indicates statistical significance ($p < 0.05$).
